# Supplementary material for: The causal relationship between the human gut microbiota and pyogenic arthritis: a Mendelian randomization study
Source: Front Cell Infect Microbiol. 2024 Nov 26;14:1452480. doi: 10.3389/fcimb.2024.1452480 (PMC11629706; doi:10.3389/fcimb.2024.1452480)
Supplement: Supplementary file 1 [file DataSheet1.zip › mendelian test/ebi-a-GCST90027726.csv_leave_one_out.pdf]

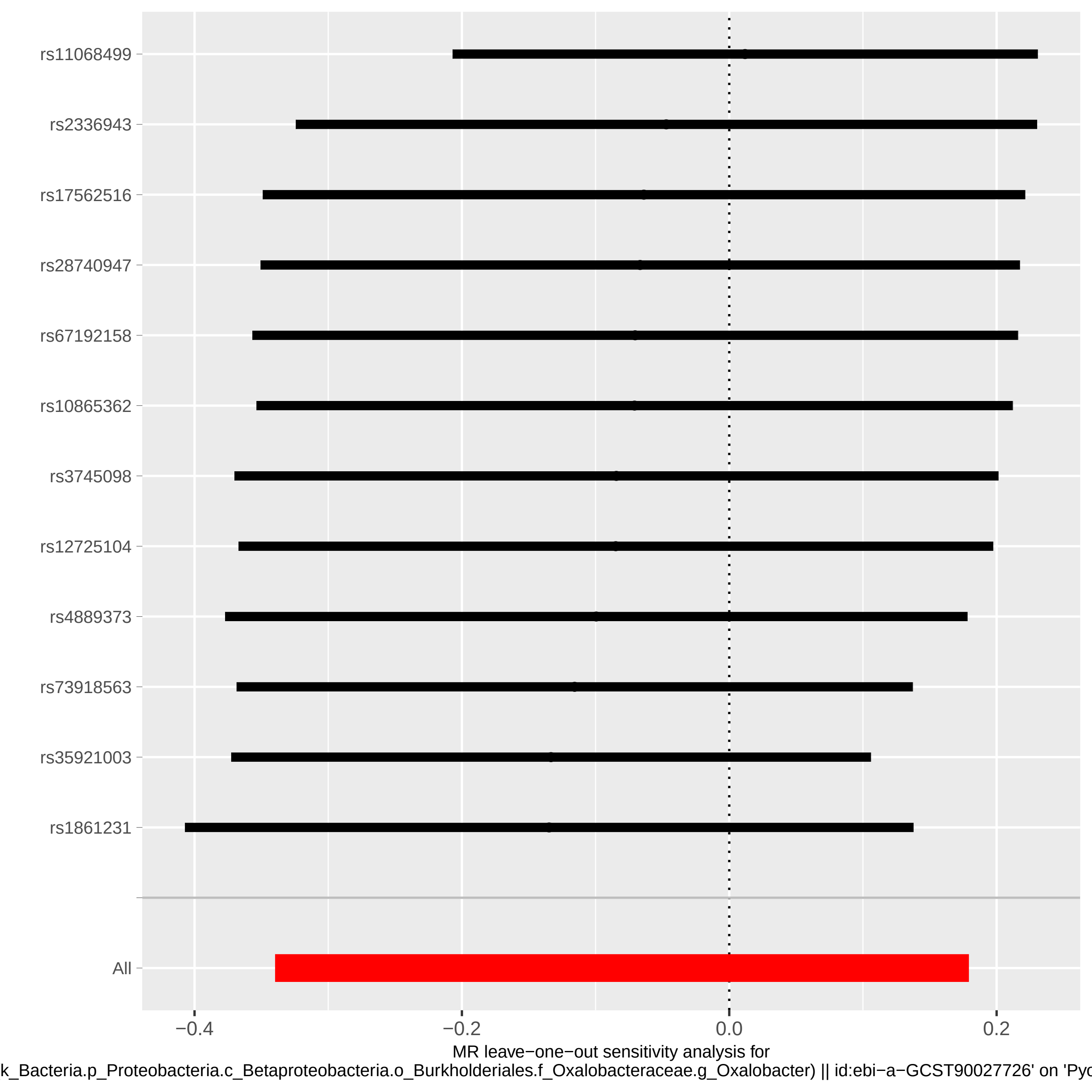
rs11068499
rs2336943
rs17562516
rs28740947
rs67192158
rs10865362
rs3745098
rs12725104
rs4889373
rs73918563
rs35921003
rs1861231
All
−0.4
−0.2
0.0
0.2
MR leave−one−out sensitivity analysis for
k_Bacteria.p_Proteobacteria.c_Betaproteobacteria.o_Burkholderiales.f_Oxalobacteraceae.g_Oxalobacter) || id:ebi−a−GCST90027726' on 'Pyo
